# Sylvian fissure development is linked to differential genetic expression in the pre-folded brain

**DOI:** 10.1038/s41598-020-71535-4

**Published:** 2020-09-02

**Authors:** Arka N. Mallela, Hansen Deng, Alyssa K. Brisbin, Alan Bush, Ezequiel Goldschmidt

**Affiliations:** 1 Department of Neurological Surgery, University of Pittsburgh Medical Center, UPMC Presbyterian Hospital, 200 Lothrop Street, Suite B-400, Pittsburgh, PA 15213 USA; 2 University of Pittsburgh School of Medicine, Pittsburgh, PA USA; 3 Department of Neurosurgery, Massachusetts General Hospital, Boston, MA USA

**Keywords:** Development of the nervous system, Neurological disorders

## Abstract

The mechanisms by which the human cerebral cortex folds into its final form remain poorly understood. With most of the current models and evidence addressing secondary folds, we sought to focus on the global geometry of the mature brain by studying its most distinctive feature, the Sylvian fissure. A digital human fetal brain atlas was developed using previously obtained MRI imaging of 81 healthy fetuses between gestational ages 21 and 38 weeks. To account for the development of the Sylvian fissure, we compared the growth of the frontotemporal opercula over the insular cortex and compared the transcriptome of the developing cortices for both regions. Spatiotemporal mapping of the lateral hemispheric surface showed the highest rate of organized growth in regions bordering the Sylvian fissure of the frontal, parietal and temporal lobes. Volumetric changes were first observed in the posterior aspect of the fissure moving anteriorly to the frontal lobe and laterally in the direction of the temporal pole. The insular region, delineated by the limiting insular gyri, expanded to a much lesser degree. The gene expression profile, before folding begins in the maturing brain, was significantly different in the developing opercular cortex compared to the insula. The Sylvian fissure forms by the relative overgrowth of the frontal and temporal lobes over the insula, corresponding to domains of highly expressed transcription factors involved in neuroepithelial cell differentiation.

## Introduction

The human cerebral cortex at birth has completed the formation of all major gyral and sulcal folds. The cerebral surface grows from a largely smooth surface beginning at the sixth month of gestation to the characteristic patterns of gyrification^1–10^. A number of hypotheses have been proposed on the mechanisms of preterm brain folding^11,12^. These include mechanical instability that can arise from the outer gray matter expanding at a faster rate than the underlying white matter, the axonal tension hypothesis in which white matter axons draw together overlying cortical regions to form gyri, and genetic prepatterning of the cortex to form convolutions^2,3,13,14^. Postnatally, cortical expansion continues through differential local growth that extend into adulthood, without altering the newborn’s brain folded outline^15^. With most of the current evidence focusing on the formation of intralobar folds, how the brain acquires its final global form is largely unknown, including the development of its most distinctive feature, the formation of the Sylvian fissure^2,3,5,13,16–18^.

Observations of cortical development in preterm infants have shown that maximal directional growth occurs from the central sulcus toward the parietal lobe, then toward the frontal and temporal regions^19,20^. The normal process of cortical development follows a predictable sequence^21^. The Sylvian fissure, the deepest sulcus on the lateral hemispheric surface, can be identified as early as 12 weeks of gestation and serves as a major landmark for the dynamic changes of the brain surface. Abnormal morphologic features of the Sylvian fissure can be frequently associated with neuronal migration disorders^22,23^. The development of this prominent fold defines the global shape of the brain and cannot be explained by current models, which render aleatory sulci and gyri, with no distinctive and reproducible large scale structure^18^.

The Sylvian fissure forms by the convergence of the frontal and temporal lobes over the insula, which is a distinctive and unique mechanism not shared by any other sulcus^24^. The characteristic radial migration pathway from the ventricular and subventricular zones, that forms most of the brain gyri, appears to be absent in the insula, which cells originate from the pallial/subpallial boundary and migrate in an oblique fashion around the basal ganglia^25^.

In the present study, our goal is to evaluate the development of the Sylvian fissure as a result of asymmetric cortical growth between the frontoparietal and temporal opercula with respect to the insular cortex. Our hypothesis is that the convergence of the frontal and temporal opercula over the insula, driven by discrete regions of high growth, are responsible for the formation of the fissure and are associated with differential genetic expression patterns.

## Results

### Differential cortical expansion and convergence drives the formation of the Sylvian fissure

**Figure 1:**
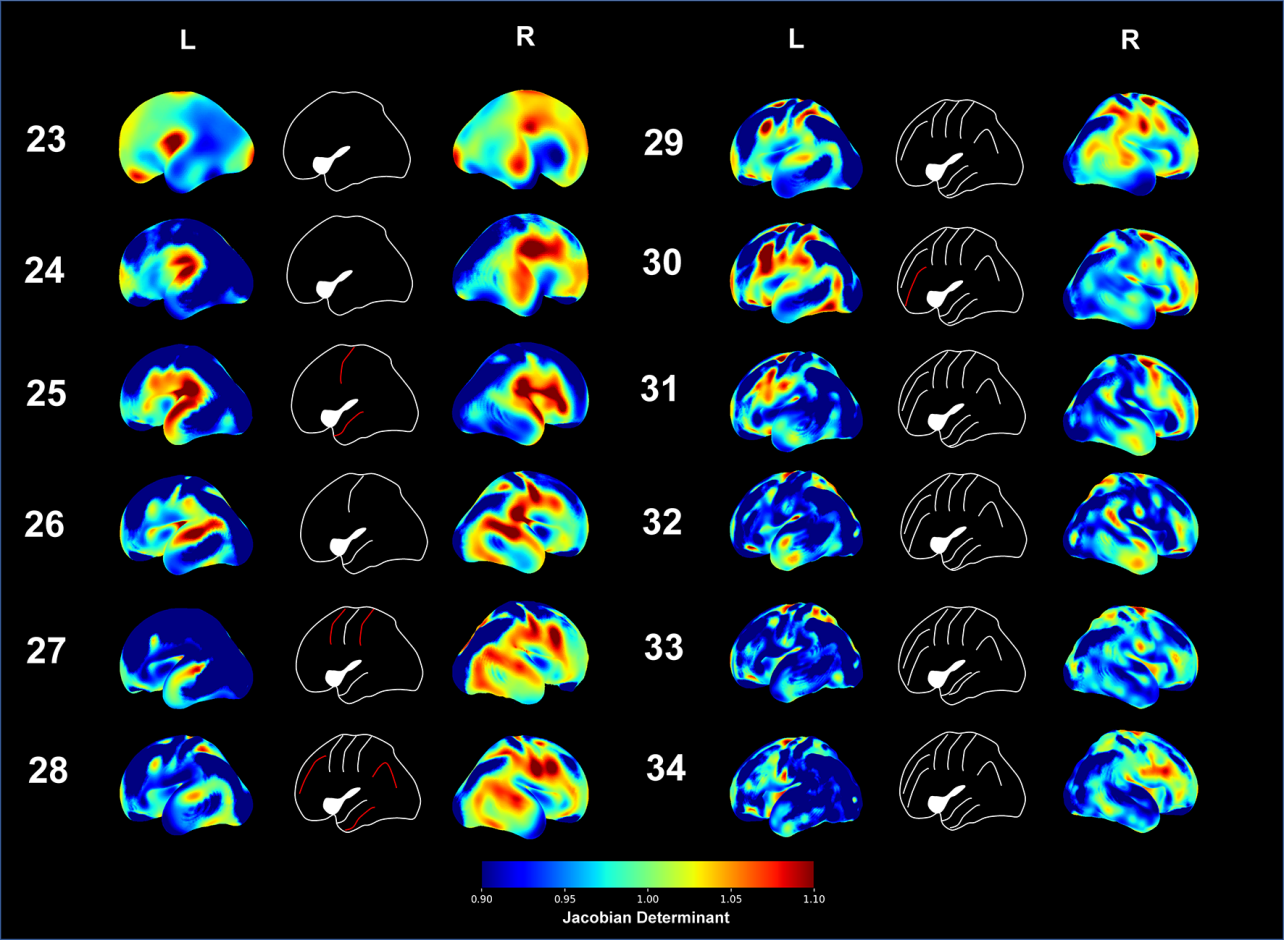
Volumetric changes on the lateral hemispheric surface during gestation. Spatiotemporal mapping of the lateral hemispheres. Blue–red color code indicates the Jacobian determinant of the week-to-week co-registration warping, a measure of local volume growth. The Jacobian determinant is normalized for global volume growth, as described in the methods. Postconceptional weeks are indicated. As can be observed, the highest rate of organized growth localizes to the opercula of insula. The first of these “hot zones” is located to the supramarginal gyrus, with the frontal and temporal poles increasing their local expansion at later times. The scale represents the weekly proportional growth (i.e. 1.1 represents a 10% weekly relative expansion). Note that week labels are gestational week (GW).

We calculated the local expansion (growth) of each area of the brain by registering each gestational week to the next one on a week by week basis. To determine local growth, we calculated the Jacobian determinant, which represents the local volumetric change (Fig. 1). This spatiotemporal mapping of the lateral hemispheres demonstrates early growth in the frontal, temporal, and parietal opercula, closing the Sylvian fissure from gestational weeks (GW) 23–25. Our analysis demonstrates that focal areas of cortical growth (volumetric expansion) and convergent growth of the opercula close the Sylvian fissure. In later weeks (GW 27–31) differential cortical expansion drives the formation of the major cortical sulci, progressing outward from the central sulcus in the frontal and parietal lobes and inferiorly in the temporal lobe. Although comparable, the left and right hemispheres exhibited asymmetric deformations and gained volume in similar places but at slightly different times, never exceeding 2 weeks of dyscoordination.

**Figure 2:**
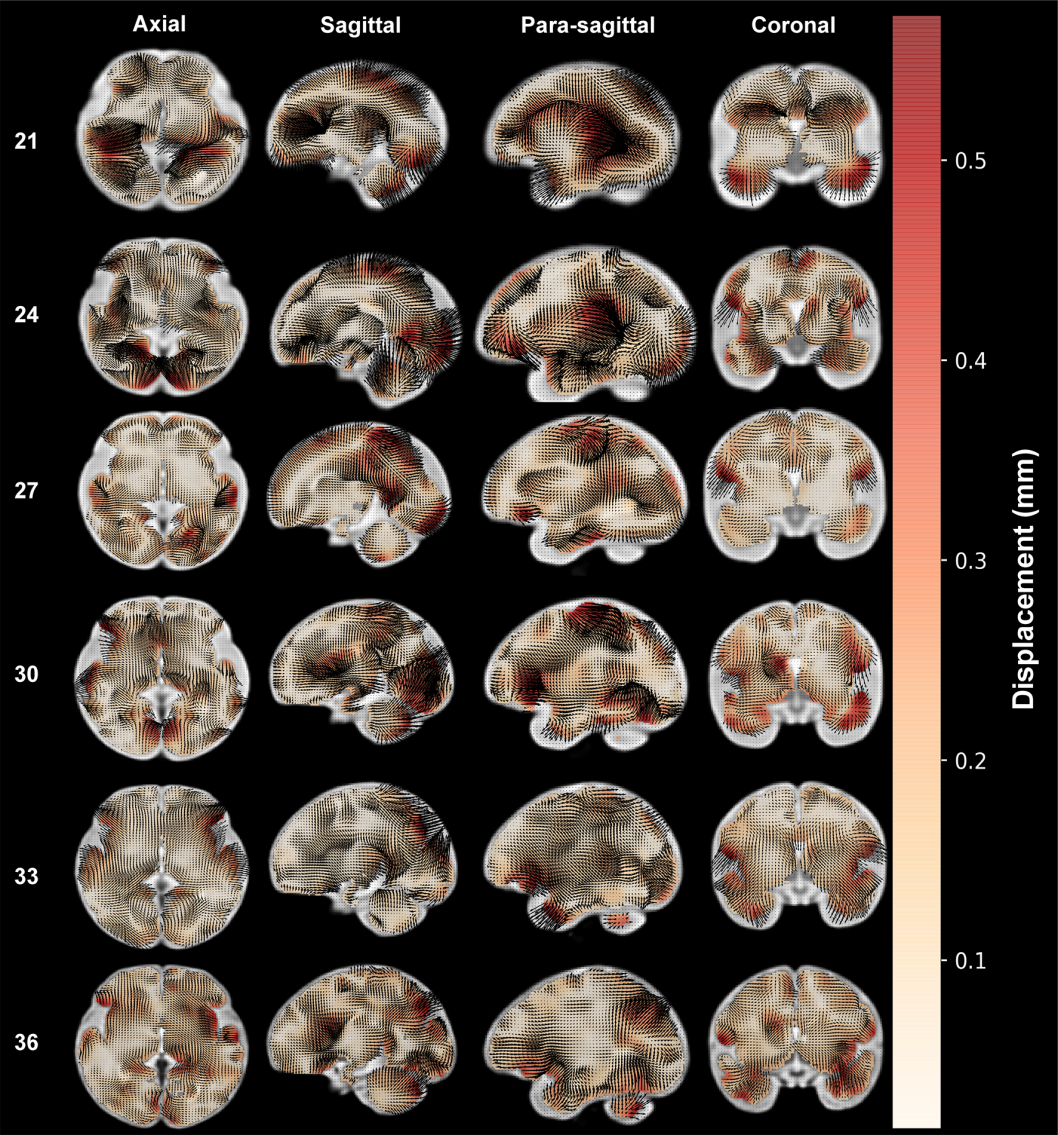
Vector displacement during development. Week-to-week displacements, calculating local tissue displacement, after normalizing for volumetric growth, were calculated for each voxel. The magnitude (total displacement) is indicated by the white to red scale (black is shading from arrows) Axial, median and coronal views at the indicated postconceptional weeks are shown. High magnitude vectors are observed close to the evolving cortex but are sparse in the white matter. The frontoparietal and temporal opercula converge over the insular cortex that is ultimately covered by them forming the Sylvian fissure. The opercula correspond to areas of high relative growth consistently throughout gestation. Note that week labels are gestational week (GW).

Using the same registration described previously, we created a vector map demonstrating the magnitude and direction of local tissue displacements (Fig. 2). This analysis demonstrates the direction of tissue growth in contrast to the Jacobian analysis which demonstrates the degree of overall tissue growth. Only cortical regions exhibited high magnitude vectors whereas the telencephalic white matter rendered neutral or small deformation levels. Both the temporal and frontotemporal opercula converged over the insula, coinciding with volumetric growth described previously. Convergence of the frontal and temporal lobes over the insula led to the formation of the Sylvian fissure. Notably, no posteroventral folding or bending was observed. This was evident at every gestational age but diminished in magnitude as the gestation progressed (Fig. 2).

### Developmental gene expression

Cortical progenitors commonly form and migrate outward from the ventricular zone by using radial glial fibers as a scaffold. As a consequence, neurons born from the same progenitor area in the ventricular zone occupy neighboring positions in the mature cortex. This is, however, not true for the insular cortex, which neurons migrate obliquely from the pallial/subpallial boundary around the basal ganglia^25^. We therefore analyzed the transcriptomes of the maturing cortices of the areas of high growth (defined by the opercula outside the insular limiting sulci, target structure) and the insula (limited by the limiting insular sulci, contrast structure). We report the top 0.1% most overexpressed genes relative to the insula, for each cellular layer of interest: subpial granular zone (SZ), marginal zone (MZ), cortical plate (CP) and subcortical plate (SP) (Table 1).

**Table 1:** Genetic expression analysis.

Layer	OMIM ID	Gene symbol	Full name	Chr	Fold change	Function	Pathological conditions	References
SG	617314	SH3YL1	SH3 domain containing, Ysc84-like 1	2	21.843	Hair follicle development, meiosis, cell migration, and dorsal ruffle formation		Blessing 2016, Hasegawa 2011
SG	605012	SUPT16H	Suppressor of Ty 16 homolog	14	20.767	Component of the FACT (facilitates chromatin transcription) complex, a chromatin-specific factor required for transcription elongation as well as for DNA replication and repair		Belotserkovskaya 2003
SG	608788	SOCS7	Suppressor of cytokine signaling 7	17	19.992	Expressed at high levels in the nervous system at embryonic day 12.5 and in the cortical plate at embryonic day 15.5	Hydrocephalus in mouse model	Krebs 2004
SG	604522	DEFA3	Defensin, alpha 3, neutrophil-specific	8	19.385	Anti-HIV activity by directly inactivating HIV particles		Mackewicz 2003
SG	609842	EDC3	Enhancer of mRNA decapping 3 homolog	15	19.23	Removal of the 5-prime cap from mRNA prior to its degradation from the 5-prime end	Mental retardation, autosomal rec. 50	Fenger-Gron 2005, Ahmed 2015
SG	N/A	ZNF563	Zinc finger protein 563	19	18.768	DNA Binding		https://www.uniprot.org/uniprot/Q8TA94
SG	600981	PGM5	Phospho-glucomutase 5	9	16.804	Phosphotransferase involved in interconversion of glucose-1-phosphate and glucose-6-phosphate		Edwards 1995
SG	609518	THAP7-AS1	THAP7 antisense RNA 1	22	16.626	Binds to N-terminal histone tails of histones H3 and H4. Promotes deacetylation (repression)		Macfarlan 2005
SG	N/A	IQUB	IQ motif and ubiquitin domain containing	7	16.172	Ubiquitin protein		https://www.genecards.org/cgi-bin/carddisp.pl?gene=IQUB
SG	610519	LOC146880	Rho GTPase activating protein 27 pseudogene	17	15.708	Involved in many cellular processes, inactive in the GDP-bound state and active in the GTP-bound stat		Katoh and Katoh 2004
SG	N/A	VSIG8		1	15.628	Immunoglobulin domain		https://www.genecards.org/cgi-bin/carddisp.pl?gene=VSIG8
SG	603560	SBF1	SET binding factor 1	22	14.732	SBF1 acts as a protective factor that prevents substrate dephosphorylation, modulates growth control	Charcot-Marie-Tooth disease, type 4B3	Cui 1998, Nakhro 2013
SG	609207	MREG	Melanoregulin	2	14.473	Melanocyte regulation		O'Sullivan 2004
SG	607753	SMUG1	Single-strand-selective monofunctional uracil-DNA glycosylase 1	12	14.45	Base excision repair—glycosylase that removes uracil from single- and double-stranded DNA in nuclear chromatin		Boorstein 2001
SG	614308	FONG		2	14.128	Unknown		Kou 2011
SG	609209	IVNS1ABP	Influenza virus NS1A binding protein	1	14.044	Actin cytoskeletal stabilization		Sasagawa 2002
SG	601534	KCNJ3	Potassium inwardly-rectifying channel, subfamily J, member 3	2	13.96	Subunit of inward-rectifying potassium channel		Kennedy 1999
SG	608214	SCN3B	Sodium channel, voltage-gated, type III, beta subunit	11	13.792	Subunit of voltage-sensitive sodium channel	Atrial fibrillation (familial, type 16), Brugada syndrome 7	Morgan 2000, Wang 2010, Hu 2009
MZ	601038	DIO3	Deiodinase, iodothyronine, type III	14	39.355	Deactivates T4/T3. Too high T4/3 can be deleterious to CNS development		Salvatore 1995
MZ	162660	NTF3	Neurotrophin 3	12	27.893	Thalamocortical connection formation, promotes the survival of, and induces neurite outgrowth from, a subset of neural crest and placode-derived neurons		Kalcheim 1992, Ma 2002
MZ	N/A	OR14C36	Olfactory receptor, family 14, subf. C, member 36	1	22.236	Olfactory receptor		https://www.genecards.org/cgi-bin/carddisp.pl?gene=OR14C36
MZ	300255	OGT	O-linked N-acetylglucosamine (GlcNAc) transferase	X	22.162	Single N-acetylglucosamine in O-glycosidic linkage to serine or threonine residues	Mental retardation, X-linked 106	Shafi 2000, Vaidyanathan 2017, Willems 2017
MZ	N/A	OR11A1	Olfactory receptor, family 11, subf. A, member 1	6	18.384	Olfactory receptor		https://www.genecards.org/cgi-bin/carddisp.pl?gene=OR11A1
MZ	612176	MYSM1	Myb-like, SWIRM and MPN domains 1	1	17.024	Metalloprotease that targets monoubiquitinated histone H2A, a mark for epigenetic transcriptional repression and chromatin inaccessibility	Bone marrow failure syndrome 4	Panda 2015, Al Sultan 2013
MZ	606198	IRX2	Iroquois homeobox 2	5	16.821	Pattern formation of vertebrate embryos		Bosse 1997
MZ	606446	SLAMF6	SLAM family member 6	1	16.416	Expressed on NK cells and cooperate in the induction of NK cell activity		Bottino 2011
MZ	602372	ZAN	Zonadhesin	7	15.498	Localizes to the anterior part of the sperm head and acts as a receptor to the zona pellucida matrix of the egg		Gasper and Swanson 2006
MZ	162660	NTF3	Neurotrophin 3	12	14.293	Thalamocortical connection formation, promotes the survival of, and induces neurite outgrowth from, a subset of neural crest and placode-derived neurons		Kalcheim 1992, Ma 2002
MZ	605799	AMN	Amnionless homolog	14	13.684	Encodes a type I transmembrane protein that is expressed exclusively in the extraembryonic visceral endoderm layer during gastrulation	Megaloblastic anemia-1, Norwegian type	Kalantry 2001
MZ	N/A	CYB561D1	Cytochrome b-561 domain containing 1	1	12.995	Cytochrome		https://www.genecards.org/cgi-bin/carddisp.pl?gene=CYB561D1
MZ	609082	FBXL16	F-box and leucine-rich repeat protein 16	16	12.441	Acts as protein-ubiquitin ligases. F-box proteins interact with SKP1 through the F box, and they interact with ubiquitination targets		Jin 2004
MZ	131550	EGFR	Epidermal growth factor receptor	7	11.919	Involved in diverse cellular functions, including cell proliferation, differentiation, motility, and survival, and in tissue development	Inflammatory skin and bowel disease, neonatal, 2; lung cancer	Wang 2004
MZ	114761	CA5A	Carbonic anhydrase VA, mitochondrial	16	11.394	Encodes an intramitochondrial carbonic anhydrase, which is pivotal for providing bicarbonate (HCO3-) for multiple mitochondrial enzymes	Hyperammonemia due to carbonic anhydrase VA deficiency	van Karnebeek 2014
MZ	612185	CASKIN2	CASK interacting protein 2	17	11.27	Binds to CASK protein, neurexins		Tabuchi 2002
MZ	N/A	TRIM4	Tripartite motif containing 4	7	10.999	Tripartite motif, localizes to cytoplasm		https://www.genecards.org/cgi-bin/carddisp.pl?gene=TRIM4
MZ	N/A	OR10J3	Olfactory receptor, family 10, subf. J, member 3	1	10.3	Olfactory receptor		https://www.genecards.org/cgi-bin/carddisp.pl?gene=OR10J3
MZ	N/A	CSAG2	CSAG family, member 2	X	10.177	Associated with chondrosarcoma		https://www.genecards.org/cgi-bin/carddisp.pl?gene=CSAG2
MZ	N/A	GRIP2	Glutamate receptor interacting protein 2	3	9.362	Interacts with Glutamate receptor		https://www.genecards.org/cgi-bin/carddisp.pl?gene=GRIP2
MZ	609626	MDGA1	MAM domain containing glycosyl phosphatidylinositol anchor 1	6	9.129	GPI-anchored membrane protein		De Juan 2002
MZ	N/A	OR5B17	Olfactory receptor, family 5, subf. B, member 17	11	8.702	Olfactory receptor		https://www.genecards.org/cgi-bin/carddisp.pl?gene=OR5B17
MZ	607419	GEMIN7	Gem (nuclear organelle) associated protein 7	19	8.537	Assembly of small nuclear ribonuculeoproteins (snRNPs). Component of Survival of Motor Neuron (SMN) Complex		Baccon 2002
MZ	607971	SLC6A15	Solute carrier family 6 (neutral amino acid transporter), member 15	12	8.475	Sodium-coupled amino acid (neurotransmitter) transporter		Takanaga 2005
MZ	607407	EBF3	Early B-cell factor 3	10	8.26	Transcription factor	Hypotonia, ataxia, and delayed development syndrome (HADDS)	Chao 2017, Harms 2017
MZ	602767	KRT85	Keratin 85	12	8.245	Keratin, component of hair follicle	Ectodermal dysplasia, Type 4	Naeem 2006
MZ	N/A	ZNF677	Zinc finger protein 677	19	8.192	Zinc finger protein (hypothetical)		https://www.genecards.org/cgi-bin/carddisp.pl?gene=ZNF677
CP	609730	PDZRN4	PDZ domain containing ring finger 4	12	5.887	Unknown		Katoh 2004
CP	602150	SNAI2	Snail homolog 2	8	5.137	SNAI2 triggers epithelial-mesenchymal transitions and plays an important role in developmental processes, evolutionarily conserved	Waardenburg syndrome type II	Perez-Mancera 2007; Sanchez-Martin 2002
CP	607047	ATXN3	Ataxin 3	14	4.983	Exhibit deubiquitinase activity and appears to be a component of the ubiquitin proteasome system. It may also have roles in transcriptional regulation and neuroprotection	Machado-Joseph disease (spinocerebellar ataxia-3)	Haacke 2006, Kawaguchi 1994
CP	608896	SGCG	Sarcoglycan, gamma (35 kDa dystrophin-associated glycoprotein)	13	4.871	The dystrophin-glycoprotein complex (DGC) comprises a group of proteins that span the sarcolemma and bind actin to the extracellular matrix of muscle cells	Muscular dystrophy, limb-girdle, autosomal recessive 5	Noguchi 1995, Piccolo 1996
CP	603054	GREM1	Gremlin 1	15	4.7	Proposed to control diverse processes in growth and development by selectively antagonizing the activities of different subsets of the transforming growth factor (TGF)-beta ligand		Hsu 1998
CP	162660	NTF3	Neurotrophin 3	12	4.604	Thalamocortical connection formation, promotes the survival of, and induces neurite outgrowth from, a subset of neural crest and placode-derived neurons		Kalcheim 1992, Ma 2002
CP	137141	GABRA4	Gamma-aminobutyric acid (GABA) A receptor, alpha 4	4	4.453	Posttranslational regulatory role of protein receptor GABRA4 subunit involved in GABAergic neurotransmission		Mu 2002
CP	123900	EZR	Ezrin	6	4.069	Scaffold between the actin cytoskeleton and transmembrane proteins facilitating cell–cell interactions and receptor retention		Roumier 2001
CP	164860	MET	Met proto-oncogene (hepatocyte growth factor receptor)	7	3.883	Cell-surface receptor for hepatocyte growth factor	Deafness, autosomal recessive 97; Hepatocellular carcinoma	Bottaro 1991
CP	615730	DOCK7	Dedicator of cytokinesis 7	1	3.86	DOCK7 plays a role in priming 1 neurite to become the axon	Epileptic encephalopathy, early infantile, 23	Watabe-Uchida 2006, Perrault 2014
CP	608789	NCKAP5	NCK-associated protein 5	2	3.858	The NAP5 protein contains pro-rich sequences and a putative nuclear localization signal. NAP5 expression was detected in fetal and adult brain, leukocytes, and fetal fibroblasts		Matuoka 1997
CP	142622	HPCA	Hippocalcin	1	3.74	Neuron-specific Ca(2+)-binding protein found in the retina and brain		Takamatsu 1994
CP	605790	STK31	Serine/threonine kinase 31	7	3.707	Encodes a putative protein kinase with a tudor domain, found in RNA-interacting proteins, and a coiled-coil domain		Wang 2001
CP	610851	AP1AR	Adaptor-related protein complex 1 associated regulatory protein	4	3.637	Membrane protein, unknown function		Simpson 2000
CP	612891	LRRC8E	Leucine rich repeat containing eight family, member E	19	3.635	Unknown		Kubota 2004
CP	601642	IL12RB2	Interleukin 12 receptor, beta 2	1	3.521	Expressed on Th1 and Th2 lymphocytes		Kim 2001
CP	N/A	OR4D6	Olfactory receptor, family 4, subf. D, member 6	11	3.48	Olfactory receptor		https://www.genecards.org/cgi-bin/carddisp.pl?gene=OR4D6
CP	606899	CACNG7	Calcium channel, voltage-dependent, gamma subunit 7	19	3.359	Component of voltage-gated calcium channel		Burgess 2001
SP	606198	IRX2	Iroquois homeobox 2	5	30.2	Pattern formation of vertebrate embryos		Bosse 1997
SP	615388	ADAT2	Adenosine deaminase, tRNA-specific 2	6	12.961	Converts adenosine to inosine by hydrolytic deamination of genomically encoded adenosine on tRNAs		Gerber 1999
SP	617922	GYPA	Glycophorin A	4	11.829	One of the most abundant red-cell proteins, with about 1 million copies of GYPA per red cell. Siaolomucin		Cooling 2015
SP	607667	CTNNA3	Catenin (cadherin-associated protein), alpha 3	10	11.647	Cell adhesion molecule. In intercalated discs of the heart, CTNNA3 is a component of a unique hybrid adhering junction, or area composita	Arrhythmogenic right ventricular dysplasia, familial, 13	Li 2012, van Hengel 2013
SP	601724	NEUROD1	Neuronal differentiation 1	2	10.769	Generate functional neurons from human pluripotent stem cells as early as 6 days after transgene activation	Maturity-onset diabetes of the young 6	Naya 1995, Pang 2011, Malecki 1999
SP	602830	HIST1H4E	Histone cluster 1, H4e	6	8.564	H4 Histone Family		Marzluff 2002
SP	601567	LMAN1	Lectin, mannose-binding, 1	18	7.921	May function as a molecular chaperone for the transport from ER to Golgi of a specific subset of secreted proteins, including coagulation factors V and VIII	Combined factor V and VIII deficiency	Nichols 1998
SP	160740	MYH2	Myosin, heavy chain 2, skeletal muscle, adult	17	7.796	Encodes the myosin heavy chain isoform that is expressed in fast type 2A muscle fibers	Proximal myopathy and ophthalmoplegia	Tajsharghi 2014
SP	118493	CHRM2	Cholinergic receptor, muscarinic 2	7	7.455	Shares structural features with other muscarinic receptors, including 7 transmembrane domains, an extracellular N terminus, and an intracellular C terminus		Peralta 1987
SP	600618	ETV6	Ets variant 6	12	7.441	May act as a tumor suppressor gene	Leukemia, acute myeloid, somatic	Stegmaier 1995
SP	608255	TRAF3IP3	TRAF3 interacting prot. 3	1	7.262	interacted with the isoleucine zipper domain of Traf3 and activated JNK		Dadgostar 2003
SP	607937	NANOG	Nanog homeobox	12	7.101	Nanog is a critical factor underlying pluripotency in both ICM and ES cells		Mitsui 2003
SP	615717	PLK1S1	Polo-like kinase 1 substrate 1	20	7.027	Mediates mitotic chromosome stabilzation	Retinitis Pigmentosa 69	Oshimori 2006, El Shamieh 2014
SP	615680	CARD16	Caspase recruitment domain fam., member 16	11	7.001	Caspase recruitment, apoptosis		Lee 2001
SP	N/A	LRRC70	Leucine rich repeat containing 70	5	7.001	Unknown		https://www.genecards.org/cgi-bin/carddisp.pl?gene=LRRC70
SP	607512	ADAMTS18	ADAM metallopeptidase with thrombospondin type 1 motif, 18	16	6.925	Zinc-dependent protease	Microcornea, myopic chorioretinal atrophy, and telecanthus	Aldahmesh 2013

Genes differentially expressed in the progenitor layers of the frontal, parietal, and temporal lobes when compared to the insula at 15 weeks post-conception in the BrainSpan atlas (https://atlas.brain-map.org/atlas?atlas=138322603) at the 99.9th percentile.

Ref: 2010 Allen Institute for Brain Science. BrainSpan Atlas of the Developing Human Brain^51^.

*SG* subpial granular Zone, *MZ* marginal Zone, *CP* cortical plate, *SP* subplate.

**Figure 3:**
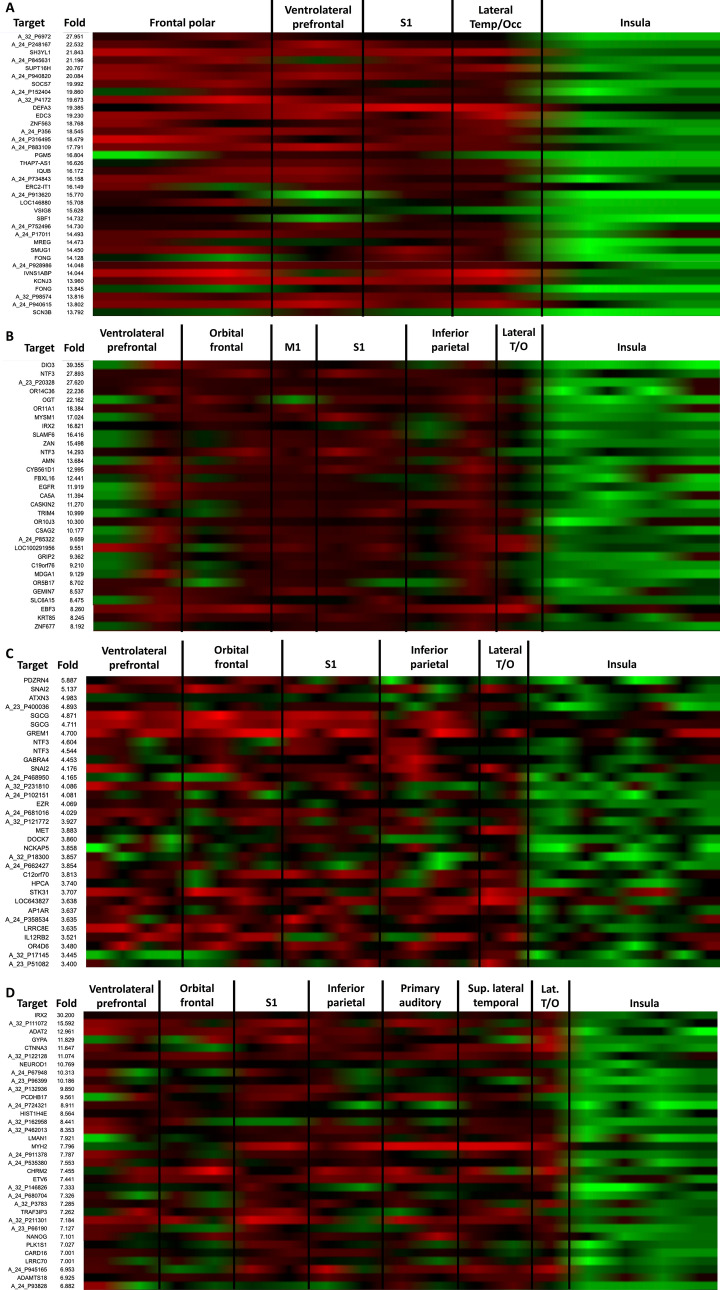
Comparison of the genetic expression in the maturing cortex of the opercula and the insula at 21 post-conceptional weeks (GW 23). A marked difference between the transcriptome of cortical areas (frontal parietal, temporal opercula) and the insular cortex. This may drive the higher degree of expansion that was observed at every level of the maturing cortex [(**A**) subpial granular zone; (**B**) marginal zone; (**C**) cortical plate and (**D**) subplate]. *M1* primary motor cortex, *S1* primary somatosensory cortex, *Lateral T/O* lateral temporooccipital. Ref: 2010 Allen Institute for Brain Science. BrainSpan Atlas of the Developing Human Brain^51^.

We found extensive differences in the transcriptome for every region, which may indicate a different origin of the cells forming these structures and may drive their differing growth rates. In the subpial granular zone, the 0.1% relative highest expressed genes had a 13.7-fold (opercula/insula) expression ratio (Fig. 3A). Amongst these, the SH3YL1 gene (related to cellular migration) and the SOCS7 gene (related to hydrocephalus in mice) are involved in CNS development. In the marginal zone the cutoff was 8.2-fold (Fig. 3B). Among the genes overexpressed in the opercular regions related to brain growth, we found DIO3, which deactivates T4 to T3 conversion; NTF3, involved in the formation of thalamocortical connections and neurite growth and survival and IRX2, involved in developmental pattern formation. For the cortical plate the fold difference to reach the 0.1 percentile was 3.4 (Fig. 3C). Several of the most overexpressed genes in this region are involved in neuronal growth including SNAI2, related to epithelial mesenchymal transitions; ATXN3, related to transcriptional regulation and neuroprotection; GREM1, proposed to control development by selectively antagonizing the activities of the transforming growth factor (TGF)-beta ligand; NTF3; GABRA4, that regulates GABAergic activity and; EZR4, that acts as a scaffold between the actin cytoskeleton and transmembrane proteins facilitating cell–cell interactions and receptor retention and DOCK7, that plays a role in priming neurites to become the axons. For the subcortical plate the threshold for reporting was 6.9 (Fig. 3D) including IRX2 and NEUROD1, that generates functional neurons from human pluripotent stem cells.

## Discussion

During the last two-thirds of gestation, the cerebral cortex expands and folds into a conserved arrangement of sulci and gyri^1–3,5^. The physical mechanisms by which the distinctive convoluted cortical pattern develops have been addressed in numerous publications^18–20^. Nevertheless, these models fail to account for the global geometry of the brain and do not explain the development of the Sylvian fissure^18–20^. Here, we found that the anterior and posterior poles of the telencephalic vesicle converge over the central region forming the frontal and temporal lobes, and the Insula, respectively. This process is triggered by the differential expansion of the fronto-temporal opercula very early during GW 23–25, preceding any bending process^22^. These two different regions exhibited a consistent difference in their transcriptomes in the pre-folded brain, with the temporal, frontal and parietal opercular cortices having consistent expression patterns that were different from that of the insula.

We interrogated the BrainSpan Atlas to determine which genes are preferentially expressed in the developing cortices of the frontal, parietal and temporal lobes compared to the insular at 21 PCW, corresponding to gestation week (GW) 23. Given the lack of longitudinal data in the BrainSpan dataset, we were not able to determine the temporal sequence of transcriptional changes that lead to folding nor the specific pathways by which these transcriptional changes effect changes in growth. Per our analysis of the Gholipour atlas, the Sylvian fissure largely closes from 23 to 25 GW, suggesting that genetic expression immediately preceding this may drive this process. Further, while MRI changes may lag anatomic changes, the closure of the Sylvian fissure is a large-scale process that is clearly visible on fetal MRI (Fig. 1). Our aim was to identify transcriptional differences at the beginning of this period (week 21) that may drive this difference in growth pattern.

Many of the overexpressed genes in the opercula have a well-defined or plausible role in neuronal migration or survival during development. SOCS7 is a protein coding gene that regulates signaling cascades and its involved in terminating neuronal migration^26^. Although the process is complex and incompletely understood, it is known that the complex SOCS7-Cul5-Rbx2 regulates termination of migration and disruption of that system results in abnormally position neurons in the CP^26^.

Similarly, Neurotrophin 3 (NTF3), IRX2, and DOCK7, have roles in neuronal differentiation and survival. NTF3 controls the survival and differentiation of neurons and is thought to promote neuronal survival in the developing brain^27,28^. In contrast, while IRX2 does not have a defined role in telencephalic development, it is known to participate in the rostro caudal differentiation of the hindbrain^29^. DOCK7 codes for a guanine nucleotide exchange factor, which in turn activates Rac 1 and 3 and Rho amongst others.DOCK7 has a direct role in brain development by regulating the fate of the radial glia^30,31^. Mutations on this gene cause epileptic encephalopathy and cortical blindness^32^.

Other proteins we identified do not have a clearly identified role (as of yet) in brain development. Ataxin 3 (ATX3) is involved in protein ubiquitination, a trinucleotide expansion of this gene (CAG) causes Machado-Joseph disease or spinocerebellar ataxia, although this mutation is associated with neuronal death, so far ATX does not have a well-defined role in brain development^33,34^. GREM1 codes for a bone morphogenetic protein (BMP) antagonist and can modulate organogenesis. In mouse it conveys the Sonic Hedgehog polarizing signal and is involved in limb development. A role in cortical folding has not been described^35^.

Apart from the physical principles involved in brain folding, numerous reports evidenced that this process is under heavy genetic regulation^5,36–40^. Recently de Juan Romero et al. described a genetic patterning in the ferret’s VZ and outer ventricular zone (OVZ), matching the folded geometry of the brain and present before gyrification started. These differentially expressed genes (DEG) are composed of thousands of sequences that are expressed in an on and off fashion alternating between sulci and gyri, including Trnp1, Ccnd1, EOMES, Notch, Shh, MAPK and Wnt^17,39,40^. This pattern conforms a blueprint for individual folds but do not explain the large-scale arrangement of the brain lobes.

A comparison between our findings and the DEGs described by de Juan Romero would be inaccurate; on one side they analyzed the ventricular and subventricular zones and not the cortices themselves; on the other, the mechanism involved in forming cortical convolutions in ferrets and humans are different and may be governed by different mechanisms^39,41,42^. The genetic expression at the pallial/subpallial boundary was described by Carney et al.^43^. This region is highly heterogeneous and expresses a mixed array of genes from the pallium (Pax6) and subpallium (Gsh2).

**Figure 4:**
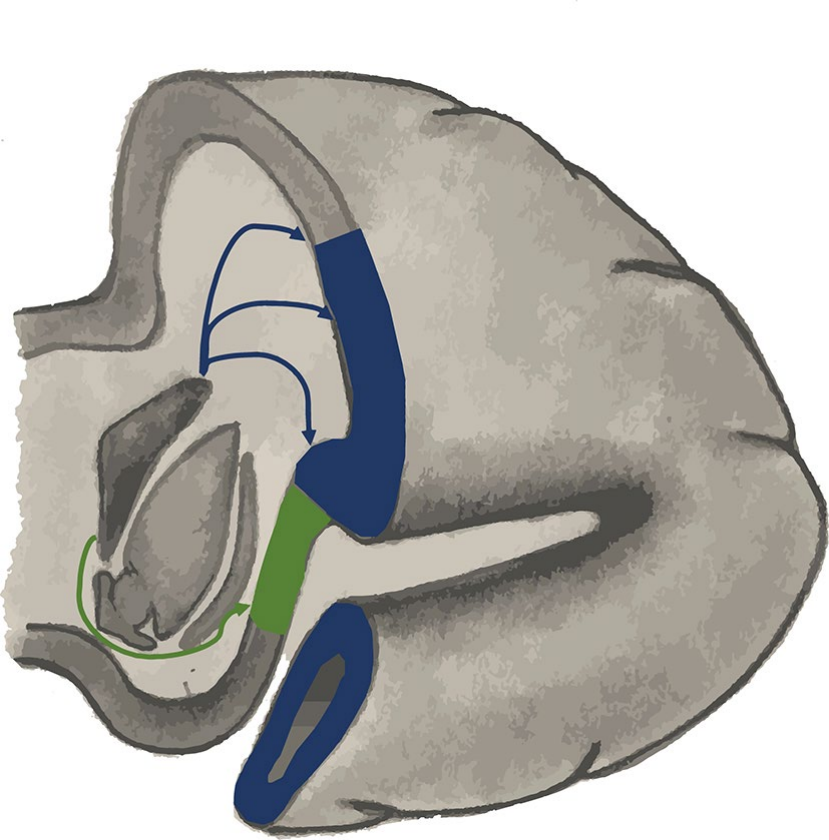
Different origins and migration paths explain the differences in growth between the insula and the frontal, parietal and temporal lobes and define the large-scale morphology of the human brain. Schematic of a 21 post-conceptional week (GW 23) brain. Radial migration from the ventricular zone (blue arrows) forms almost the entire lateral hemispheric cortical surface (blue) and is linked to the appearance of known sulci and gyri. On the other hand, the insular cortex (green) is isolated from periventricular proliferating zones by the basal ganglia and is formed by cells originating from the pallial-sub pallial boundary, defining an oblique migration pathway, which is linked with less expansion and morphologically distinct sulci.

Our findings suggest that the insular progenitor cells have a distinct transcriptional signature than the populations that develop into the frontal/temporal/parietal operculae. The latter expand at a much higher rate than the former, thus closing the Sylvian fissure on the lateral hemispheric surface. The work of de Juan Romero et al.^40^ and Carney et al.^43^ suggests that the insular progenitors may follow an oblique migration pathway in contrast to the direct radial migration pathway leading to the lateral hemispheric cortical surface (Fig. 4). The developing basal ganglia may block a direct pathway for progenitor cells in the ventricular zone to the insula. The distinct gyral configuration of the insula (5 relatively linear gyri that converge to the limen insulae) in contrast to the convoluted gyri of the lateral hemispheric surface may also be related to this^44^. Future studies must investigate the genetic makeup of these progenitor populations and how this drives the connectivity and morphology of the insula and operculae.

The atlas developed by Gholipour et al. is derived from 81 healthy, normally-developing fetuses^45^, while the gene expression data is derived from two 21 PCW (23 GW) fetuses as described in the BrainSpan documentation. It is important to note that the Gholipour atlas uses gestational week as a measure of time, which is approximately 2 weeks ahead of post-conceptional week. Thus, the genetic information from the BrainSpan Atlas corresponds to the very beginning of the Gholipour atlas. Although different individuals were used to construct both datasets, we argue that the genetic drivers of Sylvian fissure formation are highly conserved across individuals given the universal formation of the fissure in the healthy human brain.

Our findings are limited by the small number of analyzed specimens, the use of bulk mRNA instead of single cell expression, and the difficulty of matching the time and spatial dimensions between a dataset generated from MRI and one constructed directly from fetal brain tissue. Given the ethical constraints in obtaining fetal brain tissue, the number of specimens available for genetic analysis is inherently limited. Further, given these same ethical limits, longitudinal imaging and genetic analysis is not possible in the same individual. Despite these constraints, this study deepens our understanding of the geometry and the transcriptome of the normal development of the Sylvian fissure.

## Conclusion

The Sylvian fissure forms by the relative overgrowth of the frontal and temporal lobes over the insula, with the developing cortices exhibiting sharply different transcriptomes. It is interesting to note that some of these genes are related to neuronal proliferation and differentiation functions and can be part of the landscape of the genes responsible for the general geometry of the brain.

## Methods

### Guidelines statement

All data used in this analysis were obtained from publicly available databases that were obtained in accordance with all relevant guidelines and regulations. Specifically, for the fetal MRI atlas by Gholipour et al.^45^ the study was approved by the Boston Children’s Hospital Institutional Review Board and the Committee on Clinical Investigation and written informed consent was obtained from all participants. For the BrainSpan atlas, all work was performed following guidelines for the research use of human brain tissue of the University of Washington and Advanced Bioscience Resources (Alameda, California) with approval by the Human Investigation Committees and Institutional Ethics Committees of each institute.

### Developmental MRI assessment

#### Atlas

We analyzed the fetal brain atlas described by Gholipour et al.^45,46^. This atlas was reconstructed from weekly fetal T2-weighted MRI images of 81 healthy fetuses from gestational week 21 to 38 segmented to identify multiple key subcortical and cortical regions. A description of the Atlas and its full content can be accessed at https://crl.med.harvard.edu/research/fetal_brain_atlas/.

#### Registration

To analyze fetal brain development over time, we utilized the SyN algorithm in the ANTs (Advanced Normalization Tools)^47^ package to register the atlas fetal MRI on a week by week basis. This is a nonlinear, diffeomorphic registration paradigm. We performed two types of registration. First (part A), we registered images on a week-by-week basis (week 38 to week 37, week 37 to week 36, etc.) using the full SyN algorithm in the reverse direction.

Second (Part B), in order identify nonlinear changes such as gyral growth or folding and ignore gross volumetric changes, we registered all weeks to week 38 using an affine transformation (performed using ANTs). Subsequently we performed nonlinear registration using SyN to identify these nonlinear changes.

#### Registration validation

In order to validate the accuracy of our registration, we warped the week 38 segmentation to the desired week using our registration method^45^. We then compared the overlap of the atlas segmentation for the desired week to the warped segmentation using the multiclass Dice similarity coefficient, as implemented in the *Scikit-learn* package in Python.

#### Jacobian analysis

To determine volumetric changes on a week by week basis, we calculated the Jacobian determinant of Part A registration described above (week-to-week) using ANTS. The Jacobian determinant is the determinant of the Jacobian matrix, a matrix of the first order partial derivatives of the transformation, and represents the local volumetric change associated with the transformation. A determinant greater than 1 represents expansion and vice versa. We used this to generate Jacobian maps for each week representing the relative volume expansion/contraction for each voxel for each week (after correcting for global scaling). Note that the Jacobian determinants are normalized for global volume change—e.g. if overall global volume growth was 9%, an area with Jacobian determinant of 0.95 still grew 3.5%. We rendered these maps in 2D and 3D using *Nibabel, matplotlib,* and *nilearn*, all publicly available Python packages^48,49^.

#### Displacement maps

To visualize the direction and magnitude of local displacements, we rendered the normalized (part B), registration using arrow plots, which allow for visualization of local deformation^50^. In addition, we overlaid these plots with a heat map of the magnitude (total displacement) of each area. This was performed using *Nibabel* and *matplotlib*^50^.

### Developmental gene expression

#### Atlas

In the current study, the BrainSpan atlas was utilized to profile gene transcription at different stages of brain maturation. The BrainSpan atlas is an ARRA-funded grant through the NIH to a consortium consisting of the Allen Institute for Brain Science; Yale University; the University of Southern California; Massachusetts General Hospital, Harvard-MIT Health Sciences and Technology, Athinoula A. Martinos Center for Biomedical Imaging; the University of California, Los Angeles; and the University of Texas Southwestern Medical Center^51^. All data are publicly accessible. The methods and processes used to generate gene expression data have been previously published and can be accessed via https://www.brainspan.org^51^.

Postmortem human brain specimens were from the following: the Department of Neurobiology at Yale School of Medicine and the National Institute of Mental Health; the Human Fetal Tissue Repository at the Albert Einstein College of Medicine; the Brain and Tissue Bank for Developmental Disorders at the University of Maryland; the Birth Defects Research Laboratory at the University of Washington; the MRC-Wellcome Trust Human Developmental Biology Resource at the Institute of Human Genetics, University of Newcastle, U.K. The protocol was approved by the respective IRBs. Informed consent was obtained.

The BrainSpan Atlas sampled over 90 substructures in the developing brain, listed at https://help.brain-map.org/download/attachments/3506181/Prenatal_LMD_Microarray.pdf, building on the previous work by Kang et al.^7^. This includes the granular and dysgranular insular cortices (listed under “Neocortex” in Table 2 in the white paper at the link), and the surrounding opercular areas.

**Figure 5:**
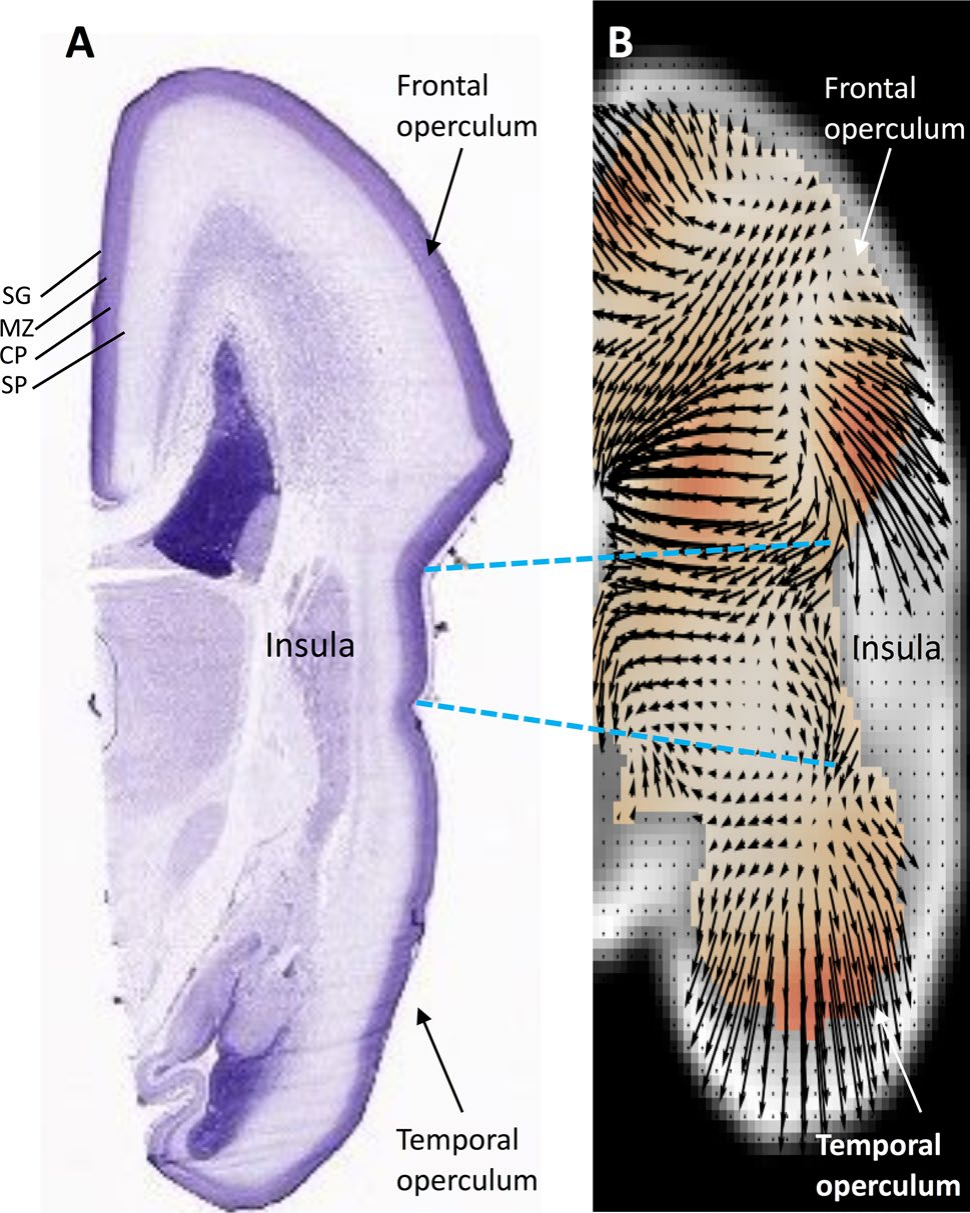
Matching areas of relatively high and slow growth with their respective transcriptomes. In order to match anatomical areas on the MR with defined regions on the transcriptome reference atlas, we referenced our analyses to constant anatomical landmarks present in both. Based on the results rendered by the MR analysis, the area within the boundaries of the limiting insular gyri, with a low relative growth rate, was compared with the fast growing opercular cortices. From superficial to deep the developing cortical regions analyzed were the subpial granular zone (SG), the marginal zone (MZ), cortical plate (CP) and the subplate (SP). The dashed blue lines connect the insular limiting sulci, which were used as reference, since they are clearly demarcated both in the genomics atlas (**A**) as well as present in our MR analysis (**B**).

**Figure 6:**
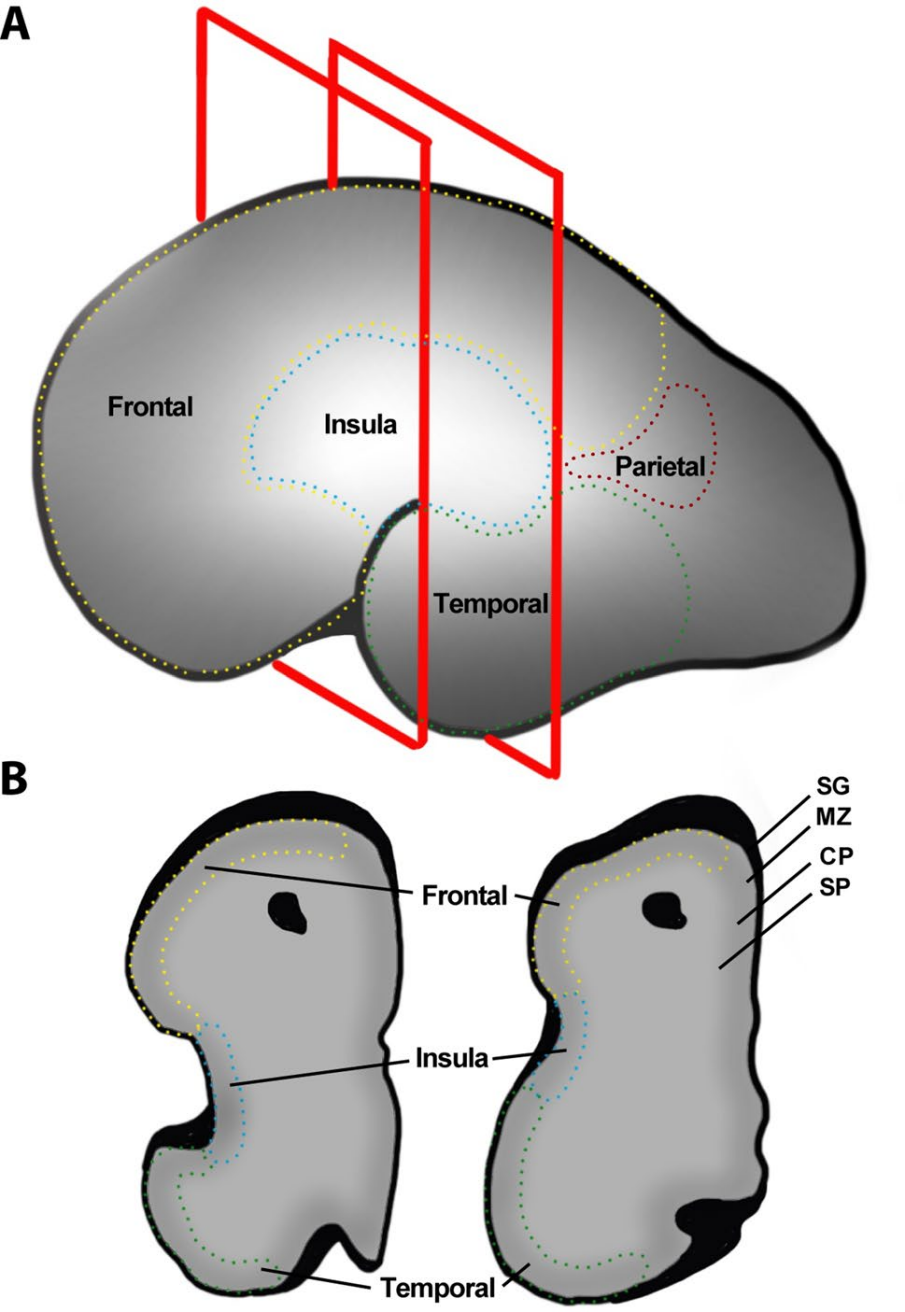
Locations of prenatal microarray profiling at PCW 21 (GW 23) and demarcations of cortical areas. Per the methodology described at the Allen Brain Atlas (https://help.brain-map.org/download/attachments/3506181/Prenatal_LMD_Microarray.pdf), the insula and surrounding operculae were specifically delineated and profiled. This figure highlights these areas—specifically for the insula (blue) and the frontal-parietal (superior—yellow) and temporal (inferior—green) operculae. The included structures from the BrainSpan atlas were—granular insular cortex, dysgranular insular cortex, frontopolar cortex, ventrolateral prefrontal cortex, orbital frontal cortex, primary motor cortex, primary somatosensory cortex, inferior parietal cortex, parainsular temporal cortex (e.g. auditory cortex), lateral temporooccipital cortex, and superior temporal cortex. (**A**) Surface illustration of the insula and operculae at PCW 21 (GW 23). The red squares indicate the locations of slices. (**B**) Coronal slices at indicated locations. These coronal slices are directly comparable to the BrainSpan atlas sections. Each region was sampled at as many as four layers—*SG* subpial granular zone, *MZ* marginal zone, *CP* cortical plate, *SP* subplate.

Because the BrainSpan Atlas transcriptome is not referenced in the stereotactic space (it is provided in coronal slices), we defined the insula and operculae with anatomical landmarks visible both in the MRI dataset and the BrainSpan Atlas. The insula was defined as the cortical region within the limiting insular sulci, which corresponded to the dysgranular and granular insular cortices in the BrainSpan Atlas. We then identified substructures in the BrainSpan atlas that correspond to the superior (frontal and parietal lobe), and inferior (temporal) operculae. Note that not all substructures had transcriptomic data for all layers (subpial granular zone, marginal zone, cortical plate, subplate). The included opercular structures from the BrainSpan atlas were—frontopolar cortex, ventrolateral prefrontal cortex, orbital frontal cortex, primary motor cortex, primary somatosensory cortex, inferior parietal cortex, parainsular temporal cortex (e.g. auditory cortex), lateral temporooccipital cortex, and superior temporal cortex. We acknowledge that several of these structures span areas beyond the conventional definition of the operculae but given the parcellation from the BrainSpan atlas, we were limited to this. This is illustrated in Figs. 5 and 6.

#### Comparative whole transcriptome analysis

We analyzed the transcriptional profile at 21 postconceptional weeks (PCW) available for a total of two specimens. We interrogated the atlas based on the result of the fetal MR analysis described above. We aimed to compare the expression profile of the developing cortex in areas of high growth with cortical regions exhibiting minimal relative expansion. The subpial granular zone (SZ), marginal zone (MZ), cortical plate (CP) and subcortical plate (SP) corresponding to the frontoparietal and temporal opercula were compared to the insula.

A comparative search of genes consistently over expressed in the target regions (opercular cortex) compared to the contrast region (insular cortex) was performed. We report on the 99.9 highest expressed percentile of around 35,000 genes analyzed for each region, which selects the top 35 overexpressed genes in each area. The cut off (in folds) was 13.7 for the SG, 8.2 for the MZ, 3.4 for the CP and 6.9 for the SP.
